# Preliminary evaluation of prostate‐targeted radiotherapy using ^131^I‐MIP‐1095 in combination with radiosensitising chemotherapeutic drugs

**DOI:** 10.1111/jphp.12558

**Published:** 2016-05-03

**Authors:** Mathias Tesson, Colin Rae, Colin Nixon, John W. Babich, Robert J. Mairs

**Affiliations:** ^1^ Institute of Cancer SciencesCollege of Medical, Veterinary and Life SciencesUniversity of GlasgowGlasgowUK; ^2^ Beatson Institute for Cancer ResearchGlasgowUK; ^3^ Division of RadiopharmacyDepartment of RadiologyCornell UniversityNew YorkNYUSA

**Keywords:** prostate‐specific membrane antigen, radiopharmaceutical, radiosensitisation, spheroids

## Abstract

**Objectives:**

Despite recent advances in the treatment of metastatic prostate cancer, survival rates are low and treatment options are limited to chemotherapy and hormonal therapy. ^131^I‐MIP‐1095 is a recently developed prostate‐specific membrane antigen (PSMA)‐targeting, small molecular weight radiopharmaceutical which has anti‐tumour activity as a single agent. Our purpose was to determine *in vitro* the potential benefit to be gained by combining ^131^I‐MIP‐1095 with cytotoxic drug treatments.

**Methods:**

Various cytotoxic agents were evaluated in combination with ^131^I‐MIP‐1095 for their capacity to delay the growth of LNCaP cells cultured as multicellular tumour spheroids. Two end‐points were used to assess treatment efficacy: (i) the time required for doubling of spheroid volume and (ii) the area under the volume–time growth curves.

**Key findings:**

The PARP‐1 inhibitor olaparib, the topoisomerase I inhibitor topotecan, the proteasome inhibitor bortezomib, the inhibitor of the P53–MDM2 interaction nutlin‐3 and the copper‐chelated form of the oxidising agent disulfiram (DSF:Cu) all significantly enhanced the inhibition of the growth of spheroids induced by ^131^I‐MIP‐1095. However, the Chk1 inhibitor AZD7762 failed to potentiate the effect of ^131^I‐MIP‐1095.

**Conclusions:**

These results indicate that targeted radiotherapy of prostate cancer may be optimised by combining its administration with chemotherapy.

## Introduction

The prognosis is favourable for prostatic carcinoma (PCa), which is restricted to the site of origin, whereas there is no cure for the disseminated disease.^1^, ^2^ While external beam radiotherapy may be effective for local control and palliation, its use to treat widespread disease is limited.^3^ Furthermore, intense local irradiation can result in significant undesirable damage to adjacent, non‐cancerous tissues, and wide‐field radiotherapy is associated with severe bone marrow toxicity. Targeted radiotherapy seeks to overcome the obstacles to cure imposed by metastatic dissemination and the intolerance of normal tissue to ionising radiation. Radiolabelled peptides based upon the glutamate–urea–lysine structure have been developed. These bind to prostate‐specific membrane antigen (PSMA) and exhibit high uptake and prolonged retention selectively in prostatic carcinoma cells and in experimental tumours.^4^, ^5^ PSMA is expressed by almost all PCa, particularly in poorly differentiated, metastatic and hormone‐refractory disease.^6^, ^7^, ^8^

The PSMA‐affinic agent, ^124^I‐MIP‐1095, detected metastatic PCa lesions in soft tissues and bone,^9^, ^10^ and recent results of the first therapeutic use of this compound labelled with iodine‐131 (^131^I) reported a reduction in bone pain and improved quality of life.^11^ It was also demonstrated that ^131^I‐MIP‐1095 did not cause immediate kidney dysfunction despite high renal uptake; only, mild and reversible haematological toxicities and xerostomia were reported.^11^ However, maximal therapeutic potency of targeted radiotherapy will be derived from its combination with radiosensitisers.^12^ Several studies have been conducted of radiosensitising agents in combination with external beam radiation. In contrast, the evaluation of drugs for the optimisation of radionuclide therapy has received relatively little scrutiny. To enhance targeted radiotherapy, there are several options for intervention, namely the DNA repair pathways, redox homoeostasis and pathways associated with pro‐ and anti‐tumour cell survival.^13^ The primary aim of this study was to determine the potential enhancement of targeted radiotherapy using ^131^I‐MIP‐1095 by means of combination with radiosensitisers (Table 1).

**Table 1:** The mechanism of action of the radiosensitisers

Drug	Mechanism of action of radiosensitisation	Target
DSF:Cu	Oxidative stress generation 43	Cellular thiols, SOD, NF‐κB
Nutlin‐	p53‐mediated apoptosis^44^	MDM2
Olaparib	Inhibition of DNA damage repair^45^, ^46^	PARP‐1
Topotecan	Generation of double‐stranded DNA breaks^47^, ^48^	Topoisomerase I
Bortezomib	Inhibition of NF‐kB activation,^49^ oxidative stress generation,^50^ downregulation of the DNA damage response^51^	26S proteasome
AZD7762	Inhibition of G_2_ arrest^35^, ^52^	Chk1

SOD, superoxide dismutase; NF‐κB, nuclear factor κB; MDM2, mouse double minute 2; PARP‐1, poly(ADP‐ribose) polymerase 1; Chk1, checkpoint kinase 1.

Evaluation of the anti‐tumour potency of DSF:Cu, nutlin‐3, olaparib, topotecan, bortezomib and AZD7762 in combination with ^131^I‐MIP‐1095 was carried out using multicellular tumour spheroids derived from the LNCaP prostate carcinoma cell line. Spheroids are representative of micrometastases in their prevascular stage of development. Radiopharmaceuticals exert their cytotoxic effect by direct deposition of energy in targeted cells and by cross‐fire to neighbouring cells. In monolayers which have accumulated radionuclides, most of the decay particle energy is deposited above and below the plane of the cultured cells. In contrast, spheroids absorb a greater proportion of cross‐fire radiation. Accordingly, spheroids constitute an appropriate model to study the therapeutic efficacy of radiopharmaceuticals. Moreover, these cellular aggregates are similar to the size class of malignant disease which is optimally sensitive to treatment with targeted radionuclides.^14^ Our findings indicate the therapeutic potential of ^131^I‐MIP‐1095 used in combination with radiosensitisers, a novel approach to the management of metastatic PCa.

## Materials and Methods

### Cell culture, drugs and solvents

The LNCaP prostate carcinoma cell line (ATCC, Middlesex, UK) was selected for its ability to internalise ^131^I‐MIP‐1095 and to grow spheroids. LNCaP cells were maintained in Roswell Park Memorial Institute medium supplemented with 10% (v/v) hyclone foetal calf serum (Fisher Scientific, Loughborough, UK), 4 mm L‐glutamine, 10 mm HEPES, 2.52 g/l D‐glucose and 1 mm sodium pyruvate. Unless otherwise stated, all reagents used for cell culture were purchased from Life Technologies (Paisley, UK). DSF, Cu and topotecan were purchased from Sigma‐Aldrich (Dorset, UK). Nutlin‐3 was purchased from Biotechne—R&D systems (Oxon, UK). Olaparib, bortezomib and AZD7762 were purchased from Stratech Scientific Ltd (Suffolk, UK). Only, topotecan and Cu were dissolved in aqueous solutions. DSF, nutlin‐3, olaparib, bortezomib and AZD7762 were dissolved in dimethyl sulfoxide (DMSO). The concentration of DMSO used for the treatment of spheroids was 0.1% (v/v). Stock solutions of drugs were prepared at 1000 times the required concentration.

### Cell cycle

LNCaP monolayers were exposed to 1 μm AZD7762 and immediately irradiated with 5 Gy using an RS225 irradiator (Xstrahl, Surrey, UK) at a dose rate of 1.64 Gy/min. After 12 h, the cells were harvested by trypsinisation and fixed in 70% ethanol at −20 °C. LNCaP cells were stained with 20 μg/ml propidium iodide and 4 μg/ml RNAse A for at least 10 min prior to analysis using FACSCalibur (BD Biosciences, Mountain View, CA), as described previously.^15^

### Spheroid initiation

LNCaP spheroids were obtained using the liquid overlay technique.^16^ The monolayers were trypsinised and reseeded at a cellular density of 120 000 cells/cm^2^ into 1% (w/v) agar‐coated flasks. After 3–4 days incubation, spheroids had formed.

### Immunohistochemistry

Hypoxia was detected using the hypoxyprobe^™^ kit (Hypoxyprobe Inc, Burlington, MA, USA). Live spheroids were treated for 24 h with 200 μm pimonidazole in culture medium prior processing. The spheroid sections were de‐waxed in xylene and re‐hydrated by successive immersions in graded alcohol and distilled water. Endogenous peroxidase activity was quenched by incubation in 0.3% (v/v) H_2_O_2_ solution in methanol for 30 min. For Ki‐67 staining only, heat‐induced antigen retrieval was performed in a 10 mm sodium citrate, 0.05% (v/v) Tween‐20, pH6 buffer at 98 °C for 25 min. The sections were then washed using Tris‐buffered Tween before being exposed to anti‐Ki67 (ThermoFisher Scientific, Hemel Hempstead, UK, 1 : 100), anti‐PSMA (DAKO, Cambridge, UK, 1 : 500) or anti‐pimonidazole adducts (Hypoxyprobe, Inc, Burlington, MA, 1 : 5000) antibodies. For pimonidazole and PSMA staining, the secondary antibody was the rabbit biotinylated polyclonal anti‐mouse antibody (DAKO, Cambridge, UK, 1 : 100). For Ki67 staining, the anti‐rabbit EnVision^™^ system (DAKO, Cambridge, UK) was used in conjunction with the 3, 3′‐diaminobenzidine (DAB) substrate kit for peroxidase (Vector Laboratories, Burlingame, CA). Spheroid sections were counterstained with haematoxylin and immersed in Scott's tap water. The haematoxylin and eosin (H&E) staining procedure involved successive immersions of spheroid sections in haematoxylin, 1% (v/v) acid alcohol, Scott's tap water and eosin with intermediate washes in water. Finally, the sections were dehydrated and rewaxed by successive immersions in graded alcohol and xylene before mounting on a glass coverslip.

### ^131^I‐MIP‐1095 radiolabelling

^131^I‐MIP‐1095 was synthesised as described previously.^5^ Briefly, radiolabelling was accomplished by iododestannylation of the trimethylstannyl precursor with 1.85–3.7 GBq of sodium iodide‐^131^ using acidic oxidising conditions to form ^131^I‐MIP‐1095 in moderate radiochemical yield (50–70%). The radioiododestannylation afforded the ^131^I‐labelled tri‐*tert*‐butyl esters that were purified using C18 Sep Pak columns and deprotected with trifluoroacetic acid to afford the desired radioiodinated compound in >95% radiochemical purity. The specific activity was determined to be ≥ 148 GBq/μmol.

### ^131^I‐MIP‐1095 uptake assay

LNCaP spheroids were incubated for a range of times in culture medium containing 0.37 MBq/ml ^131^I‐MIP‐1095. LNCaP spheroids were then washed three times in culture medium. The radioactivity retained in the spheroids was measured using a γ‐counter (Canberra Packard, Berkshire, UK). Protein extracts of spheroids were obtained by incubation in 100 μl lysis buffer (protease inhibitor (Calbiochem), 1.19 g HEPES, 1.46 g NaCl and 0.5 ml Nonidet P‐40 in 100 ml distilled water, pH 7) for 45 min on ice. Protein concentration was determined using the Bradford assay.^17^ Radiopharmaceutical uptake was expressed as counts per minute (CPM) per mg of protein.

### Treatment of spheroids and growth curve analysis

LNCaP spheroids were treated with ^131^I‐MIP‐1095 for 2 h in the presence or absence of various radiosensitisers. Thereafter, excess ^131^I‐MIP‐1095 was removed by washing. The spheroids were re‐incubated for 22 h in the presence of radiosensitisers before their removal by washing. Then, spheroids of approximately 100 μm in diameter were manually selected and individually transferred into agar‐coated wells. Two orthogonal diameters, d_max_ and d_min_ (μm), were measured using the image analysis software ImageJ, and the volume, V (10^6^ μm^3^), was calculated using: V = π × d_max _× d_min_²/6 000 000.^18^ To enable comparison between treatments, the volume, V, of a single spheroid was divided by its initial volume V_0_ (V/V_0_). Linear regression analysis of the relationship between the logarithm of the V/V_0_ value and time t was performed using the method of least squares. The linear regression equation was fitted to the exponential part of the spheroid growth curve. The slope, b, and the y‐intercept, a, of the linear regression equation logV/V_0 _= bt + a were used to calculate the time, τ_2_, required for a two‐fold increase in spheroid volume from day 0: τ_2 _= (log2−a)/b. To evaluate the effect of combination treatment over the whole course of the experiment, the area under the logV/V_0_ vs time curve (AUC) was also calculated for individual spheroids using trapezoidal approximation.

### Statistical analysis

Statistical analyses were carried out using the software SPSS v.19 (IBM, New York, NY, USA). The distributions of τ_2_ and AUC values were not normal, as indicated by the Shapiro–Wilk test. Therefore, nonparametric Kruskal–Wallis testing was used to determine whether experimental data indicated a significant level of difference between the medians of the groups. If the *P*‐value of the Kruskal–Wallis test was <0.05, the Mann–Whitney test was used for pairwise comparisons. Firstly, to demonstrate enhancement of radiation‐induced spheroid growth delay by radiosensitisers, the observed effect in response to a combination treatment of a radiosensitiser with ^131^I‐MIP‐1095 had to be concomitantly greater than that induced by ^131^I‐MIP‐1095 alone and than that induced by the radiosensitiser alone. Secondly, an absence of enhancement of ^131^I‐MIP‐1095‐induced growth delay could be due to insufficient radiosensitiser dosage. Therefore, the evaluation of the modification of the effect of ^131^I‐MIP‐1095 involved a family of four pairwise comparisons: radiosensitiser vs untreated control, ^131^I‐MIP‐1095 vs untreated control, radiosensitiser + ^131^I‐MIP‐1095 vs radiosensitiser and radiosensitiser + ^131^I‐MIP‐1095 vs ^131^I‐MIP‐1095. To compensate for multiple pairwise comparisons, Bonferroni correction was applied. To retain the criterion *P *<* *0.05, the level of significance of each pairwise comparison was set to 0.0125.

## Results

### Morphological characterisation of LNCaP spheroids

**Figure 1:**
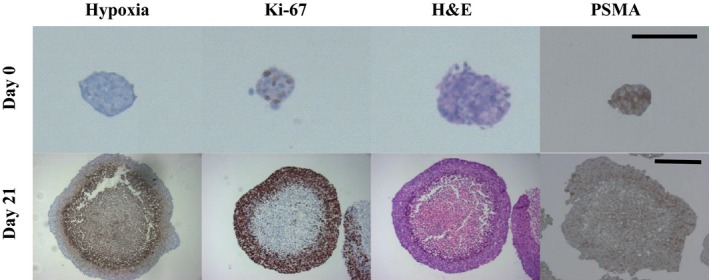
The effect of size on internal LNCaP spheroid morphology. The markers of hypoxia (pimonidazole adducts), proliferation (Ki‐67) and histological organisation (haematoxylin and eosin, H&E) as well as PSMA were detected by immunohistochemistry in LNCaP spheroid sections 0 and 21 days following initiation. The bars indicate 150 μm.

The effect of size on the internal morphology of LNCaP spheroids was analysed. H&E staining revealed a size‐dependent change in LNCaP spheroid internal structure (Figure 1). LNCaP spheroids of approximately 100 μm diameter were oxygenated and contained Ki‐67‐positive, proliferating cells homogeneously distributed throughout the section (Figure 1). In contrast, mature LNCaP spheroids of approximately 500 μm in diameter contained a hypoxic core surrounded by an outer layer of Ki‐67‐positive proliferating cells (Figure 1). In both spheroids of 100 and 500 μm in diameter, PSMA expression was homogeneous throughout the sections, regardless of internal morphology (Figure 1). LNCaP spheroids, as models of avascular micrometastasis and of approximately 100 μm diameter, were selected for the investigation of ^131^I‐MIP‐1095 uptake and growth delay.

### Characterisation of the spheroid growth delay induced by ^131^I‐MIP‐1095

**Figure 2:**
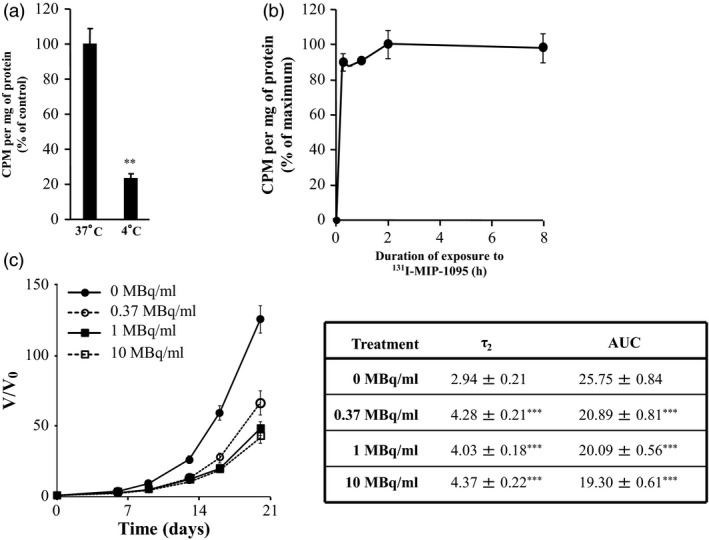
The time dependency of ^131^I‐MIP‐1095 binding to LNCaP spheroids. (a) The effect of temperature on uptake following treatment with 0.37 MBq/ml ^131^I‐MIP‐1095 for 2 h. Independent *t*‐test: **P < 0.01. (b) The binding kinetic of 0.37 MBq/ml ^131^I‐MIP‐1095 to LNCaP spheroids was evaluated at 4 °C. (c) The growth of LNCaP spheroids following treatment with ^131^I‐MIP‐1095 was evaluated. The τ_2_ and AUC values were calculated to quantify spheroids growth delay according to material and methods. The medians of τ_2_ and AUC values of the groups treated with ^131^I‐MIP‐1095 alone were statistically compared with that of the untreated controls (*). Data are mean ± SEM (*n* = 3), ***P < 0.001.

The temperature‐dependent and PSMA‐specific internalisation of ^131^I‐MIP‐1095 by LNCaP cells grown as monolayers is an endocytotic process.^4^ At 4 °C, the association of ^131^I‐MIP‐1095 with spheroids was 25% of that obtained at 37 °C (*P *<* *0.01) (Figure 2a). The uptake observed at 4 °C represents the fraction of ^131^I‐MIP‐1095 bound to PSMA, whereas cellular accumulation at 37 °C is the sum of binding and internalisation of radiopharmaceutical. This apportionment of activity in spheroids is similar to that previously observed in cellular monolayers.^4^ Saturation of binding of ^131^I‐MIP‐1095 to PSMA at 4 °C was achieved after 2 h (Figure 2b).

Based on the uptake data, the effect of ^131^I‐MIP‐1095 treatment for 2 h on the growth of LNCaP spheroids was evaluated as a single agent in order to determine the radioactive concentration to be used in combination with radiosensitisers (Figure 2c). There was a significant modification of τ_2_ and AUC values in response to treatment with ^131^I‐MIP‐1095. For instance, the AUC values decreased from 25.75 ± 0.84 to 20.89 ± 0.81, 20.09 ± 0.56 or 19.30 ± 0.61 in response to treatment with 0, 0.37, 1 or 10 MBq/ml ^131^I‐MIP‐1095. Correspondingly, τ_2_ values increased from 2.94 ± 0.21 to 4.28 ± 0.21, 4.03 ± 0.18 or 4.37 ± 0.22 days in response to treatment with 0, 0.37, 1 or 10 MBq/ml ^131^I‐MIP‐1095 (Figure 2c). Based on these results, a radioactivity concentration of 0.37 MBq/ml ^131^I‐MIP‐1095 was selected for the assessment of the modulation of radiopharmaceutical‐induced growth delay.

### Evaluation of the potential enhancement of the spheroid growth delay induced by ^131^I‐MIP‐1095 by combination with radiosensitisers

The selection of radiosensitising concentrations of drugs was based on previously published data derived from experimentation *in vitro* as well as plasma concentrations achieved in clinical trials (Table 2).

**Table 2:** Comparison between plasma concentrations and *in vitro* radiosensitising concentrations of the drugs

Drug	Radiosensitising concentration	Plasma concentration
DSF:Cu	1 μm	1 μm 19
Topotecan	10 nm	7.21–17.03 nm 20 24.8–108.9 nm 25
Olaparib	1 μm	0.23–2.3 μm 24 5.75 μm 23 11 μm 24
Bortezomib	10 nm	580 nm 21 205.60 nm 22

**Figure 3:**
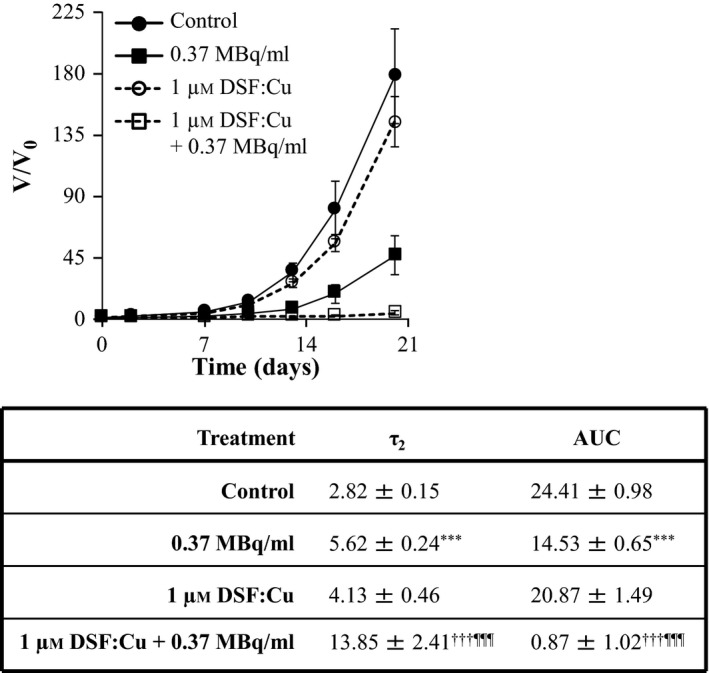
The effect of DSF:Cu on the growth delay induced by ^131^I‐MIP‐1095 in LNCaP spheroids. The effect of DSF:Cu on the growth delay induced by 0.37 MBq/ml ^131^I‐MIP‐1095 was evaluated in LNCaP spheroids. The medians of τ_2_ and AUC values of the spheroids exposed to the single agent treatments were statistically compared with that of the untreated controls (*). The medians of τ_2_ and AUC values of the spheroids treated with a combination of ^131^I‐MIP‐1095 with DSF:Cu were compared with those of the spheroids treated with ^131^I‐MIP‐1095 alone (¶) and to those of the spheroids treated with DSF:Cu alone (†). Data are mean ± SEM (*n* = 3), and three symbols indicate *P* < 0.001.

The enhancement of ^131^I‐MIP‐1095 spheroid growth delay by DSF:Cu was demonstrated by the statistically significant modulation of τ_2_ and AUC values (Figure 3). For instance, the AUC values were 14.53 ± 0.65 (*P < *0.001), 20.87 ± 1.49 and 0.87 ± 1.02 (*P *<* *0.001) for spheroids treated with 0.37 MBq/ml ^131^I‐MIP‐1095 alone, 1 μm DSF:Cu alone and the combination of both agents, respectively. The analogous τ_2_ values were 5.62 ± 0.24 (*P < *0.001), 4.13 ± 0.46 and 13.85 ± 2.41 (*P *<* *0.001) days for spheroids treated with 0.37 MBq/ml ^131^I‐MIP‐1095 alone, 1 μm DSF:Cu alone and the combination of both agents, respectively (Figure 3).

**Figure 4:**
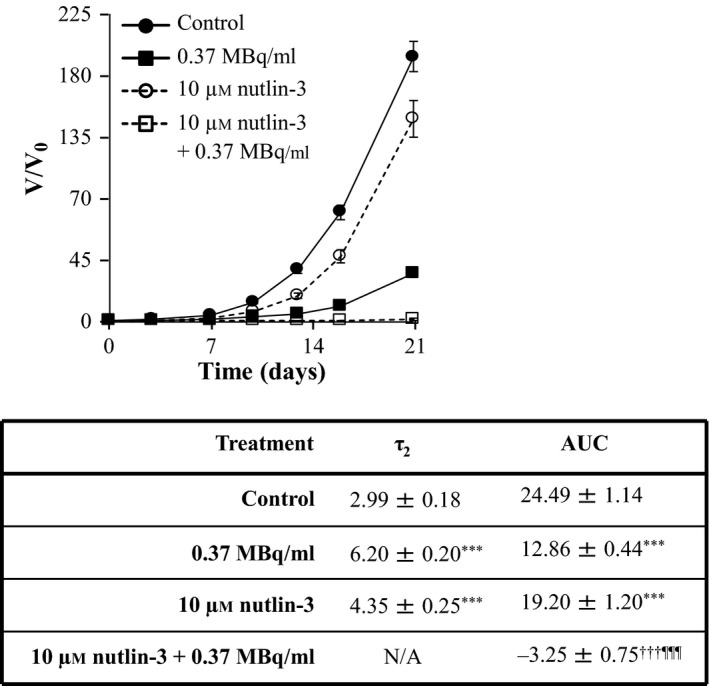
The effect of nutlin‐3 on the growth delay induced by ^131^I‐MIP‐1095 in LNCaP spheroids. The effect of nutlin‐3 on the growth delay induced by 0.37 MBq/ml ^131^I‐MIP‐1095 was evaluated in LNCaP spheroids. The medians of τ_2_ and AUC values of the spheroids exposed to the single agent treatments were statistically compared with that of the untreated controls (*). The medians of τ_2_ and AUC values of the spheroids treated with a combination of ^131^I‐MIP‐1095 with nutlin‐3 were compared with those of the spheroids treated with ^131^I‐MIP‐1095 alone (¶) and to those of the spheroids treated with nutlin‐3 alone (†). Data are mean ± SEM (*n* = 3), and three symbols indicate *P* < 0.001.

**Figure 5:**
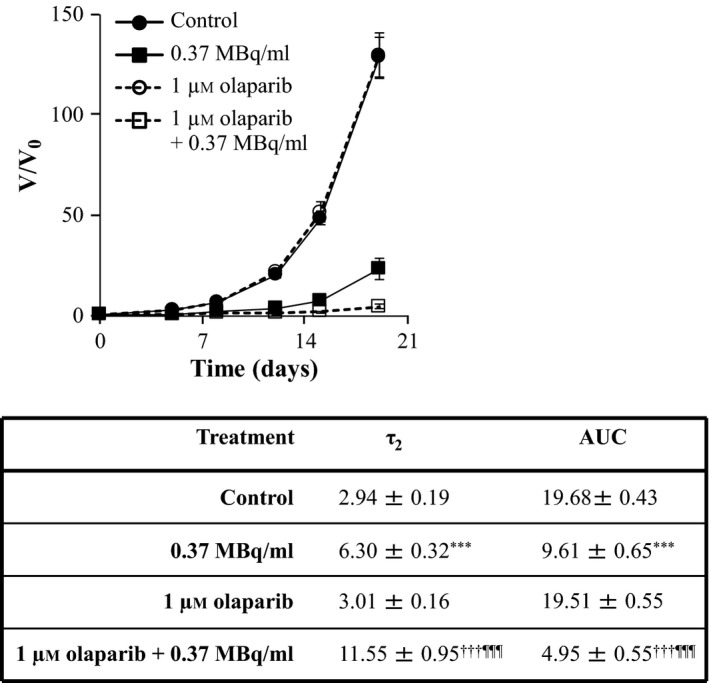
The effect of olaparib on the growth delay induced by ^131^I‐MIP‐1095 in LNCaP spheroids. The effect of olaparib on the growth delay induced by 0.37 MBq/ml ^131^I‐MIP‐1095 was evaluated in LNCaP spheroids. The medians of τ_2_ and AUC values of the spheroids exposed to the single agent treatments were statistically compared with that of the untreated controls (*). The medians of τ_2_ and AUC values of the spheroids treated with a combination of^ 131^I‐MIP‐1095 with olaparib were compared with those of the spheroids treated with ^131^I‐MIP‐1095 alone (¶) and to those of the spheroids treated with olaparib alone (†). Data are mean ± SEM (*n* = 3), and three symbols indicate *P* < 0.001.

**Figure 6:**
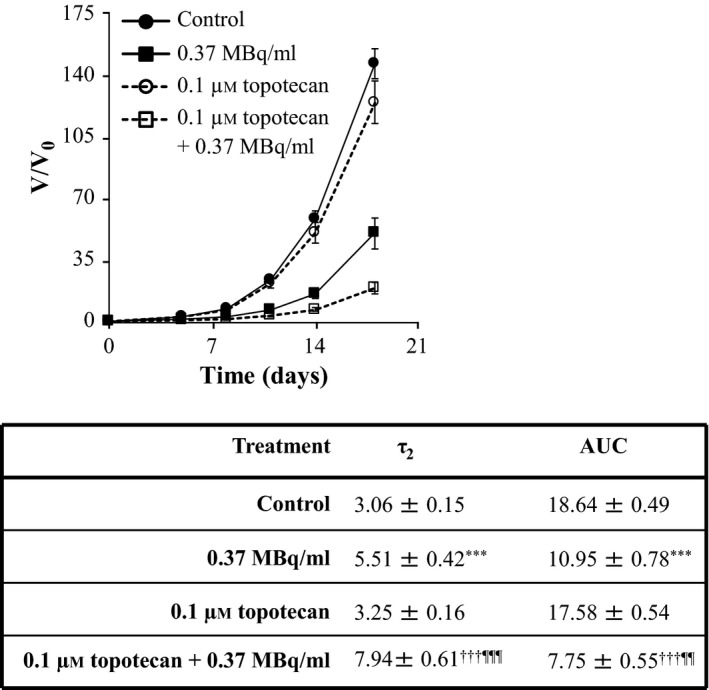
The effect of topotecan on the growth delay induced by ^131^I‐MIP‐1095 in LNCaP spheroids. The effect of topotecan on the growth delay induced by 0.37 MBq/ml ^131^I‐MIP‐1095 was evaluated in LNCaP spheroids. The medians of τ_2_ and AUC values of the spheroids exposed to the single agent treatments were statistically compared with that of the untreated controls (*). The medians of τ_2_ and AUC values of the spheroids treated with a combination of ^131^I‐MIP‐1095 with topotecan were compared with those of the spheroids treated with ^131^I‐MIP‐1095 alone (¶) and to those of the spheroids treated with topotecan alone (†). Data are mean ± SEM (*n* = 3), two symbols indicate *P* < 0.01, and three symbols indicate *P* < 0.001.

**Figure 7:**
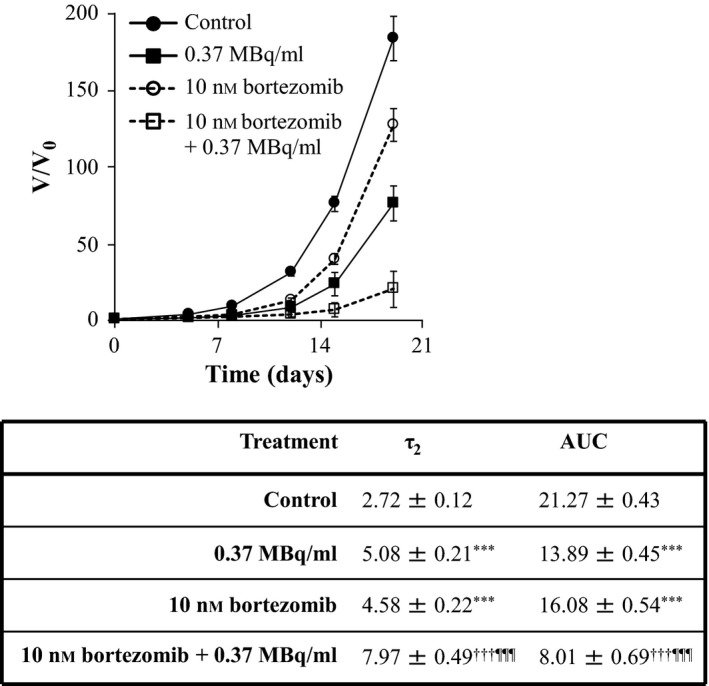
The effect of bortezomib on the growth delay induced by ^131^I‐MIP‐1095 in LNCaP spheroids. The effect of bortezomib on the growth delay induced by 0.37 MBq/ml ^131^I‐MIP‐1095 was evaluated in LNCaP spheroids. The medians of τ_2_ and AUC values of the spheroids exposed to the single agent treatments were statistically compared with that of the untreated controls (*). The medians of τ_2_ and AUC values of the spheroids treated with a combination of ^131^I‐MIP‐1095 with bortezomib were compared with those of the spheroids treated with ^131^I‐MIP‐1095 alone (¶) and to those of the spheroids treated with bortezomib alone (†). Data are mean ± SEM (*n* = 3), three symbols indicate *P* < 0.001.

Similarly, the enhancements of ^131^I‐MIP‐1095 spheroid growth delay by 10 μm nutlin‐3 (Figure 4), 1 μm olaparib (Figure 5), 0.1 μm topotecan (Figure 6) and 10 nm bortezomib (Figure 7) were indicated by the statistically significant modulation of τ_2_ and AUC values. For nutlin‐3, the determination of τ_2_ values was impossible because the spheroids exposed to the combination treatment did not double in size (Figure 4).

**Figure 8:**
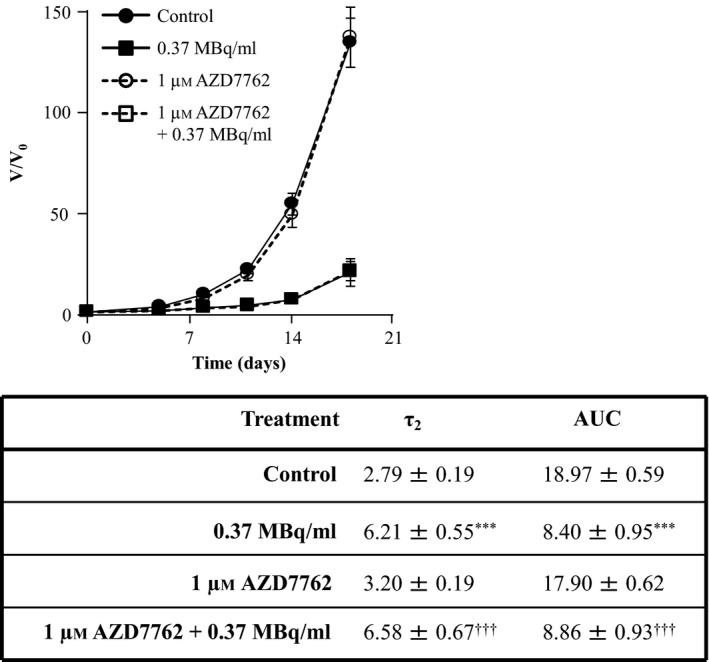
The effect of AZD7762 on the growth delay induced by ^131^I‐MIP‐1095 in LNCaP spheroids. The effect of AZD7762 on the growth delay induced by 0.37 MBq/ml ^131^I‐MIP‐1095 was evaluated in LNCaP spheroids. The medians of τ_2_ and AUC values of the spheroids exposed to the single agent treatments were statistically compared with that of the untreated controls (*). The medians of τ_2_ and AUC values of the spheroids treated with a combination of ^131^I‐MIP‐1095 with AZD7762 were compared with those of the spheroids treated with ^131^I‐MIP‐1095 alone (¶) and to those of the spheroids treated with AZD7762 alone (†). Data are mean ± SEM (*n* = 3), three symbols indicate *P* < 0.001.

**Figure 9:**
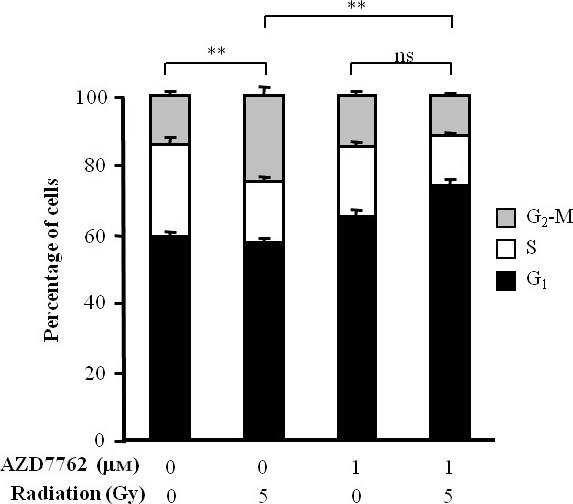
The effect of 1 μm AZD7762 on the γ‐radiation‐induced G2 arrest. The distribution of LNCaP cells throughout the cell cycle was determined by fluorescence‐activated cell sorting of propidium iodide‐stained cells 12 h following irradiation with 5 Gy in the presence of 1 μm AZD7762. One‐way ANOVA with Bonferroni correction was used to compare the mean percentage in G2‐M. Data are mean ± SEM (*n* = 3), **P < 0.01, and ns indicates *P* > 0.05.

Treatment with 1 μm AZD7762 as a single agent did not result in spheroid growth delay nor did it enhance the spheroid growth delay induced by ^131^I‐MIP‐1095 (Figure 8). For instance, the AUC values were 8.40 ± 0.95 (*P < *0.001), 17.90 ± 0.62 and 8.86 ± 0.93 following treatment with 0.37 MBq/ml ^131^I‐MIP‐1095 alone, 1 μm AZD7762 alone and the combination of both agents, respectively. Similarly, the τ_2_ values were 6.21 ± 0.55 (*P < *0.001), 3.20 ± 0.19 and 6.58 ± 0.67 following treatment with 0.37 MBq/ml ^131^I‐MIP‐1095 alone, 1 μm AZD7762 alone and the combination of both agents, respectively (Figure 8). Furthermore, treatment with 1 μm AZD7762 prevented the accumulation of LNCaP cells in G_2_ following γ‐radiation treatment (Figure 9). The proportions of LNCaP cells in G_2_ were 14.67% ± 0.98, 25.90% ± 2.46, 16.00% ± 0.93 and 13.30% ± 0.51 for the untreated group and those treated with 5 Gy, 1 μm AZD7762 and the combination of both agents, respectively. This result suggested that the lack of sensitisation to ^131^I‐MIP‐1095 cannot be attributed to a subeffective dosage of AZD7762.

## Discussion

Our purpose was to evaluate the potency of radiosensitisers, with various mechanisms of action, in combination with the PSMA‐specific radiopharmaceutical ^131^I‐MIP‐1095. Modulators of the DNA damage response are represented by nutlin‐3, which induces p53‐mediated apoptosis, olaparib, which inhibits DNA repair and the G_2_ arrest inhibitor AZD7762. The oxidative agent DSF:Cu, the DNA replication poison topotecan and the proteasome inhibitor bortezomib were also assessed. A comparison of the radiosensitising potency of drugs which possess a variety of biological actions—for example DNA repair inhibitors vs cell cycle regulation inhibitor vs ROS generators—is not meaningful in the absence of knowledge of the cancer phenotype of the target population. For instance, G_2_ arrest inhibitors are most appropriate for the treatment of p53^−/−^ cancer types with efficient DNA repair capacity, whereas the administration of nutlin‐3 may be more suitable for the management of p53^+/+^ cancer types. Furthermore, a comparison of the radiosensitising effectiveness of drugs is only possible at equipotent concentrations of radiosensitisers as single agents, that is at concentrations which inhibit the target to the same extent.

Drugs which counteract stress responses such as DNA damage repair inhibitors or modifiers of the cell cycle response to radiation treatment are especially attractive for use in combination with targeted radiopharmaceuticals as the absence of a cytotoxic effect as a single agent would spare non‐malignant tissues which do not accumulate tumour‐targeted radiopharmaceutical. Therefore, the choice of a radiosensitiser depends on the radiosensitising potential, the phenotype of the cancer and on the toxicity profile. Furthermore, it is important that the drug concentrations shown to enhance the growth delay induced by ^131^I‐MIP‐1095 are clinically relevant. It has been shown that the radiosensitising concentrations of DSF:Cu, olaparib, topotecan and bortezomib described in this report are achievable in the plasma of patients (Table 2).^19^, ^20^, ^21^, ^22^, ^23^, ^24^, ^25^, ^26^ Currently, there is no phase I or II clinical trials involving nutlin‐3.^27^, ^28^

While it is expected that the most significant influence of treatment with radiosensitisers will be potentiation of the damage inflicted by decay particle bombardment of susceptible cellular elements, it is noteworthy that enhancement of therapeutic efficacy of radiopharmaceutical may also result from increased cellular uptake. Indeed, pretreatment with topotecan,^29^ cisplatin^30^ or doxorubicin^30^ has been reported to enhance the accumulation of tumour‐targeted radiopharmaceuticals. However, in the foregoing studies, prior incubation for 24–48 h was required for enhancement of cellular uptake, whereas no corresponding pretreatment was applied in the current study. Therefore, simultaneous exposure to ^131^I‐MIP‐1095 and cytotoxic drugs is unlikely to have resulted in increased intracellular concentration, suggesting that radiosensitisation may be the sole mechanism modulating ^131^I‐MIP‐1095‐induced inhibition of spheroid growth. However, the evaluation of alternative schedules of delivery of ^131^I‐MIP‐1095 and radiosensitisers is required to derive maximal therapeutic efficacy.

We observed that the PARP‐1 inhibitor olaparib potentiated the LNCaP spheroid growth delay induced by ^131^I‐MIP‐1095. Significantly, targeted radiotherapy is delivered at a markedly lower dose rate than external beam radiation.^31^ Moreover, it has been shown that PARP inhibitors are especially effective in the enhancement of radiation kill at low doses.^32^ Therefore, PARP inhibitors may be appropriate for combination with targeted radiopharmaceuticals characterised by a low dose‐rate radiation.

We report no enhancement of ^131^I‐MIP‐1095‐induced spheroid growth delay by the Chk1 inhibitor AZD7762. This may be due to LNCaP cells harbouring a functional p53 pathway,^33^, ^34^ which is known to limit the radiosensitising potential of G_2_ arrest inhibitors such as AZD7762.^35^ Furthermore, it has been suggested that the radiosensitivity of LNCaP cells may be due to inefficient DNA damage repair in G_2_ phase.^36^ Therefore, the observed decrease in the duration of G_2_ arrest induced by AZD7762 following irradiation, which results in a reduction of the extent of DNA damage repair, may be of no consequence. These observations highlight the importance of the knowledge of the genotypic characteristics of tumours, for the selection of radiosensitiser–radiopharmaceutical combinations.

Spheroids which grow to a diameter of approximately 300 μm develop a non‐proliferative, hypoxic core surrounded by a proliferative layer of cells.^37^ These features of spheroids in an advanced stage of growth confer resistance to therapy.^38^, ^39^, ^40^ The current study of the modulation of growth delay induced by ^131^I‐MIP‐1095 was conducted using spheroids of 100 μm diameter which had not yet undergone internal morphological changes. In agreement with previous reports,^41^, ^42^ we observed that PSMA expression was homogeneous in small spheroids. However, larger spheroids of 500 μm in diameter were also characterised by uniform expression of PSMA. It is recommended that the current study should be developed to address the potentiating effect of the cytotoxic drugs on the growth delay induced by ^131^I‐MIP‐1095 in spheroids which have developed a hypoxic core surrounded by proliferating region of cells. Such analyses should evaluate the ability of combinations of therapeutic modalities to overcome the anticipated resistance to treatment conferred by non‐proliferative and hypoxic regions.

## Conclusions

Our preliminary screening indicates that the disruption of the cell cycle (topotecan, bortezomib, nutlin‐3), the generation of oxidative stress (DSF:Cu) or the inhibition of DNA repair (olaparib) are mechanisms which may be exploited to enhance the anti‐tumour potency of ^131^I‐MIP‐1095. Further investigation *in vivo* of the efficacy of radiosensitisers in combination with ^131^I‐MIP‐1095 may expedite progress of this therapeutic strategy for the clinical management of metastatic PCa.

## Declarations

### Conflict of interest

The authors declare that they have no conflict of interest to disclose.

### Funding

This work was supported by generous grants from Prostate Cancer UK, Children with Cancer UK, Action Medical Research and Neuroblastoma UK.
